# Open transinguinal preperitoneal mesh repair of inguinal hernia: a targeted systematic review and meta-analysis of published randomized controlled trials

**DOI:** 10.1093/gastro/got002

**Published:** 2013-04-05

**Authors:** Muhammad S. Sajid, L. Craciunas, K.K. Singh, P. Sains, M.K. Baig

**Affiliations:** Department of General & Laparoscopic Colorectal Surgery, Worthing Hospital, Worthing. West Sussex. BN11 2DH. UK

**Keywords:** inguinal hernia, transinguinal preperitoneal mesh repair, Lichtenstein repair, chronic groin pain

## Abstract

**Objective:** The objective of this article is to systematically analyse the randomized, controlled trials comparing transinguinal preperitoneal (TIPP) and Lichtenstein repair (LR) for inguinal hernia.

**Methods:** Randomized, controlled trials comparing TIPP vs LR were analysed systematically using RevMan® and combined outcomes were expressed as risk ratio (RR) and standardized mean difference.

**Results:** Twelve randomized trials evaluating 1437 patients were retrieved from the electronic databases. There were 714 patients in the TIPP repair group and 723 patients in the LR group. There was significant heterogeneity among trials (*P* < 0.0001). Therefore, in the random effects model, TIPP repair was associated with a reduced risk of developing chronic groin pain (RR, 0.48; 95% CI, 0.26, 0.89; z = 2.33; *P* < 0.02) without influencing the incidence of inguinal hernia recurrence (RR, 0.18; 95% CI, 0.36, 1.83; z = 0.51; *P* = 0.61). Risk of developing postoperative complications and moderate-to-severe postoperative pain was similar following TIPP repair and LR. In addition, duration of operation was statistically similar in both groups.

**Conclusion:** TIPP repair for inguinal hernia is associated with lower risk of developing chronic groin pain. It is comparable with LR in terms of risk of hernia recurrence, postoperative complications, duration of operation and intensity of postoperative pain.

## INTRODUCTION

Mesh repair of inguinal hernia is the most common operation performed on general surgical patients. Approximately 20 million groin hernioplasties are performed each year worldwide, over 17 000 operations in Sweden, over 12 000 in Finland, over 80 000 in England and over 800 000 in the USA [1–4]. Countless studies have been reported in the medical literature in attempts to improve the overall outcomes following hernia operations and, due to this fact, the procedure has evolved immensely, especially over the last few decades. Recurrence of inguinal hernia was initially a significant problem; `however, with the advent of the tension-free mesh repair as described as Lichtenstein repair (LR) [5], recurrence rate has consistently been reported as low as 1–4% [6–10], a drop from up to 50–60%. Concomitant with this drop in the hernia recurrence rate, investigators and surgeons are facing other challenges, such as an increased incidence of chronic pain following LR. There are several controversies regarding definition of chronic pain but a relatively accepted definition has been put forth by the International Association for the Study of Pain and cited by Poobalan *et al.* [11], is pain that persists at the surgical site and nearby surrounding tissues beyond 3 months. However, persistence of surgical site pain at six months after surgery is also reported in few studies. Incidence of postoperative chronic groin pain ranges from 10–54% of patients following inguinal hernia operation [11–13]. The exact mechanism involved in the development of chronic groin pain following LR and laparoscopic inguinal hernia repair is still poorly understood but it is postulated to be multifactorial in origin. The etiological factors leading to post-operative chronic groin pain include inguinal nerve irritation by the sutures or mesh [14], inflammatory reactions against the mesh [15] or simply scarring in the inguinal region incorporating the inguinal nerves [16–18]. It may also be attributed to local tissue inflammatory reactions from foreign material, bio-incompatibility and abdominal wall compliance reduction [19]. In addition, fixation of the mesh during LR and laparoscopic inguinal hernia repair is thought to contribute to postoperative chronic groin pain due to nerve injury ranging from 2–4% [20].

Transinguinal preperitoneal (TIPP) inguinal hernia repair with soft mesh has been reported as a safe anterior approach with a preperitoneal sutureless mesh position by using the annulus internus as an entrance to the preperitoneal space [21–23]. This open and sutureless technique has a short learning curve and it is also cost-effective compared to the laparoscopic total extraperitoneal preperitoneal technique [24]. Theoretically, TIPP repair may be associated with lesser chronic postoperative pain than Lichtenstein’s technique due to the placement of mesh in the preperitoneal space to avoid direct regional nerves dissection and their exposure to bio-reactive synthetic mesh. The placement of mesh in this plane without using any suture for fixation—and lack of mesh exposure to regional nerves—was assumed to result in the reduced risk of developing chronic groin pain. A recently published Cochrane review of two published and one unpublished randomized, controlled trials failed to provide adequate evidence in favour of TIPP repair due to lack of an optimum number of studies and recruited patients [25]. In addition, another recently reported meta-analysis of 12 studies (10 randomized, controlled trials and two comparative studies) confirmed the potential benefits of TIPP in terms of reduced risk of developing chronic groin pain with equivocal postoperative complications and risk of hernia recurrence [26]. This meta-analysis also failed to provide a conclusive statement because it included trials comparing LR against the Prolene™ Hernia System. Therefore, the objective of this review article is to systematically analyse the randomized, controlled trials comparing TIPP and LR of inguinal hernia with mesh and attempt to ascertain the role of TIPP in reducing the incidence of chronic groin pain without influencing the risk hernia recurrence and postoperative complications.

## METHODS

### Identification of trials

Randomized, controlled trials (irrespective of language, country of origin, hospital of origin, blinding, sample size or publication status) comparing TIPP vs LR approaches of open inguinal hernia repair were included in this review. We also included other trials in which mesh was placed in the preperitoneal space through an open inguinal incision approach. The Cochrane Colorectal Cancer Group (CCCG) Controlled Trials Register, the Cochrane Central Register of Controlled Trials (CENTRAL) in the Cochrane Library, Medline, Embase and Science Citation Index Expanded were searched for articles published up to October 2012 using the medical subject headings (MeSH) terms “inguinal hernia” and “groin hernia” in combination with free text search terms, such as “mesh repair of inguinal hernia”, “transinguinal preperitoneal repair”, “sutureless repair” and “open inguinal hernia repair”. A filter for identifying randomized, controlled trials recommended by the Cochrane Collaboration was used to filter out non-randomized studies in Medline and Embase [27]. The references from the included trials were searched to identify additional trials.

### Data extraction

Two of the authors independently identified the trials for inclusion and exclusion and extracted the data. The accuracy of the extracted data was further confirmed by a third author. There were no discrepancies in the selection of the trials or in data extraction between the reviewers, except in the case of recording the severity of pain according to the measurement scales and timing of the recorded data. All reviewers agreed that blinding was impossible to achieve in the case of the operating surgeon. However, there was disagreement with regard to whether the trials should be classified as having a high or low risk of bias, based on four parameters, i.e. randomization technique, power calculations, blinding and intention-to-treat analysis. It was agreed that the lack of an adequate randomization technique and an intention-to-treat analysis would result in the trials being classified as having a high risk of bias. In case of any unclear or missing information, the reviewers planned to obtain those by contacting the authors of the individual trials.

### Statistical analysis

The software package RevMan 5.1.2 [28], provided by the Cochrane Collaboration, was used for the statistical analysis to achieve a combined outcome. The risk ratio (RR) with a 95 per cent confidence interval (CI) was calculated for binary data and the standardized mean difference (SMD) with a 95% CI was calculated for continuous data variables. The random-effects model was used to calculate the combined outcomes of both binary and continuous data [29, 30]. Heterogeneity was explored using the chi-squared test, with significance set at *P* < 0.05 and was quantified using I^2^ [31], with a maximum value of 30% identifying low heterogeneity [31]. The Mantel-Haenszel method was used for the calculation of RR under the random effect models [32]. In a sensitivity analysis, 0.5 was added to each cell frequency for trials in which no event occurred in either the treatment or control group, according to the method recommended by Deeks *et al.* [33]. If the standard deviation was not available, then it was calculated according to the guidelines of the Cochrane Collaboration [27]. This process involved assumptions that both groups had the same variance, which may not have been true, and variance was either estimated from the range or from the *P*-value. The estimate of the difference between both techniques was pooled, depending upon the effect weights in results determined by each trial estimate variance. A forest plot was used for the graphical display of the results. The square around the estimate stood for the accuracy of the estimation (sample size) and the horizontal line represented the 95% CI. The methodological quality of the included trials was initially assessed using the published guidelines of Jadad *et al.* and Chalmers *et al.* [34, 35]. Based on the quality of the included randomised, controlled trials, the strength and summary of the evidence was further evaluated by GradePro® [36], a tool provided by the Cochrane Collaboration. We classified chronic groin pain and recurrence as primary outcome measures. Duration of operation, postoperative pain and postoperative complications were analysed as secondary outcome measures.

## RESULTS

**Figure 1:**
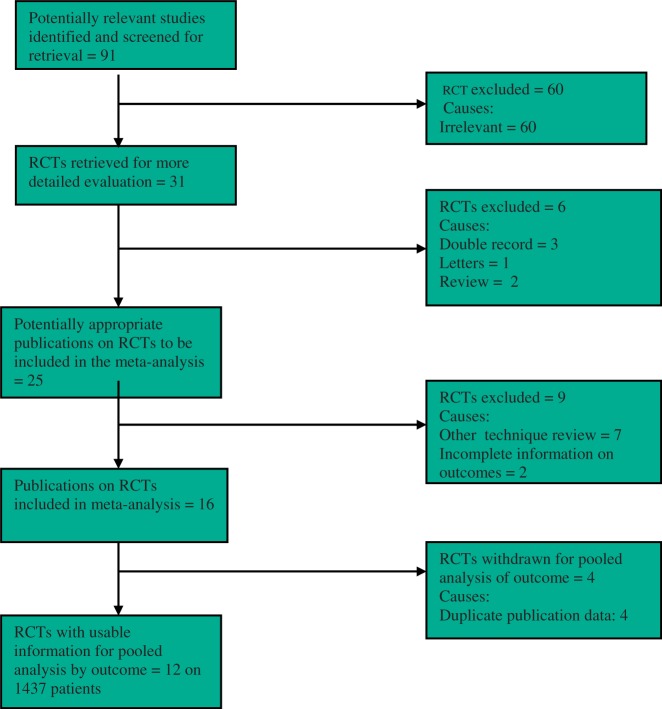
PRISMA flow chart showing trial selection methodology.

The PRISMA flow chart to explain the literature search strategy and trial selection is given in Figure 1. Twelve randomized, controlled trials evaluating 1437 patients were retrieved from commonly used standard medical electronic databases [37–48]. There were 714 patients in the TIPP repair group and 723 patients in the LR group. The characteristics of the included trials are given in Table 1. The salient features and treatment protocols adopted in the included randomized, controlled trials are given in Table 2. The short summary of data, selected primary and secondary outcome measures used to achieve a summated statistical effect, are given in Table 3. Three included trials [41, 42, 48] reported four study arms but their data pertaining to TIPP repair and LR was used exclusively for this analysis. Similarly we used data of TIPP repair and LR arm from another trial which reported five study arms [44]. We also included a trial [43] which recruited acute surgical patients of incarcerated inguinal undergoing TIPP repair vs LR. Subgroup analysis after excluding these trials favoured the principle conclusion of this review.

**Table 1:** Characteristics of included trials

Trial	Year	Country	Age in years	Male: Female	Duration of follow-up	Hernia details
Berrevoet *et al.* [37]
TIPP	2012	Belgium	18–65	142: 8	3 months	Primary inguinal hernia
LR						
Dogru *et al.* [38]					months	
TIPP	2006	Turkey	51.1 ± 16.2	134: 5	53.06 ± 5.6	Primary inguinal hernia
LR			50.1 ± 16.4		53.41 ± 7.1	
Erhan *et al.* [39]
TIPP	2008	Turkey	58.9 (36–82)	Males only	12 months	Primary inguinal hernia
LR			57.1 (17–85)			Recurrent inguinal hernia
Farooq *et al.* [40]
TIPP	2007	Pakistan	56.7	Males only	24 months	Primary inguinal hernia
LR						Recurrent inguinal hernia
Gunal *et al.* [41]
TIPP	2007	Turkey	23.85 ± 0.49	Males only	96 months	Primary inguinal hernia
LR			22.76 ± 0.3			
Hamza *et al.* [42]
TIPP	2010	Egypt	35.67 ± 12.96	Males only	12 months	Primary inguinal hernia
LR			35.12 ± 10.11			
Karatepe *et al.* [43]
TIPP	2008	Turkey	63 ± 20.1	31: 9	6–72 months	Primary inguinal hernia
LR			60 ± 17.7		6–70 months	Recurrent inguinal hernia
Kawji *et al.* [44]
TIPP	1999	Austria	57–72	Mixed group	18 months	Primary inguinal hernia
LR (5 arms)			65			
Koning *et al.* [45]
TIPP	2012	Netherlands	57 ± 12.1	288: 14	12 months	Primary inguinal hernia
LR			56.5 ± 13.2			
Muldoon *et al.* [46]
TIPP	2004	USA	60.7 (26–86)	Males only	24 months	Primary inguinal hernia
LR			63.3 (18–85)			
Nienhuijs *et al.* [47]
TIPP	2007	Netherlands	55.6 ± 15.8	170: 2	3 months	Primary inguinal hernia
LR			54 ± 13.6			
Vatansev *et al.* [48]
TIPP	2002	Turkey	50.7 ± 15.7	40: 5	1 week	Primary inguinal and femoral
LR			53.2 ± 12.6			hernia

TIPP = transinguinal preperitoneal hernia repair, LR = Liechtenstein repair.

**Table 2:** Treatment protocol adopted in included trials

Trial	Transinguinal preperitoneal hernia repair	Lichtenstein repair
Berrevoet *et al.* [37]	Transinguinal preperitoneal hernia repair Mesh details not available Mesh fixation technique not available	Standard LR of inguinal hernia Mesh and fixation technique not available
Dogru *et al.* [38]	Same incision and approach as in LR Kugel’s method for mesh placement Kugel’s mesh was used No mesh fixation reported	Standard LR of inguinal hernia 6 x 11 cm Prolene mesh was used No mesh fixation technique reported
Erhan *et al.* [39]	Same incision and approach as in LR 15 x 15 cm Prolene mesh was used Mesh fixed with 00 Prolene stitch in preperitoneal space	Standard LR of inguinal hernia Prolene mesh was used Mesh fixed with 0 Prolene stitch
Farooq *et al.* [40]	Preperitoneal space was entered through transverse lower abdominal incision 3 cm above the inguinal ligament Same mesh and fixation suture as in LR	Standard LR of inguinal hernia Prolene mesh was used Mesh fixed with 0 Prolene stitch
Gunal *et al.* [41]	Nyhus preperitoneal approach 6 x 12 cm Prolene mesh was used Mesh and fixation technique not reported	Standard LR of inguinal hernia 6 x 12 cm Prolene mesh was used Mesh and fixation technique not reported
Hamza *et al.* [42]	Standard TIPP repair of inguinal hernia Mesh and fixation technique not reported	Standard LR of inguinal hernia Mesh and fixation technique not reported
Karatepe *et al.* [43] TIPP LR	Standard TIPP repair of inguinal hernia 10 x 15 cm Prolene mesh was used No fixation of the mesh	Standard LR of inguinal hernia Prolene mesh was used Mesh fixed with 0 Prolene stitch
Kawji *et al.* [44]	Wantz TIPP repair of inguinal hernia Mesh and fixation technique not reported	Standard LR under LA and GA Mesh and fixation technique not reported
Koning *et al.* [45]	Standard TIPP repair of inguinal hernia Polysoft™ mesh 16 x 9.5 cm with memory ring was used No mesh fixation	Standard LR of inguinal hernia SoftMesh™ 6 x 13.7 cm was used Mesh fixed with 3/0 Prolene stitch
Muldoon *et al.* [46]	Read-Rives preperitoneal approach 12 x 16 cm Prolene mesh was used Mesh fixed with 2/0 Prolene stitch	Standard LR of inguinal hernia 7 x 15 cm Prolene mesh was used Mesh fixed with 2/0 Prolene stitch
Nienhuijs *et al.* [47]	Kugel’s method for mesh placement Kugel’s mesh 11 x 14 cm was used No mesh fixation reported	Standard LR of inguinal hernia 6 x 11 cm Prolene mesh was used Mesh fixed with non-absorbable suture
Vatansev *et al.* [48]	Nyhus preperitoneal approach Mesh and fixation technique not reported	Standard LR of inguinal hernia Mesh and fixation technique not reported

TIPP = transinguinal preperitoneal hernia repair, LR = Liechtenstein repair, LA = local anaesthetic, GA = general anaesthetic.

**Table 3:** Variables used for meta-analysis

Trial	Patients (number: *n*)	Operation time (minutes ± SD)	Perioperative pain: 30 day (*n*)	Complications (*n*)	Chronic groin pain (*n*)	Recurrence (*n*)
Berrevoet *et al.* [37]^1^
TIPP	75	Not available	5/75	2/75	2/72	3/72
LR	75		29/75	14/75	10/56	2/70
Dogru *et al.* [38]
TIPP	69	45.36 ± 6.20	Not investigated	5	0	0/69
LR	70	47.06 ± 7.50		2	0	1/70
Erhan *et al.* [39]
TIPP	24	Not investigated	Not investigated	1	1	1
LR	70			0	4	0
Farooq *et al.* [40]
TIPP	33	62.6 ± 18.4*	Not investigated	1	0	0
LR	34	70.1 ± 18.4		6	0	0
Gunal *et al.* [41]^2^
TIPP	39	36.54 ± 1.55	3.7 ± 1	9	0	1/39
LR	42	39.64 ± 1.28	4.8 ± 1.4	19	0	1/42
Hamza *et al.* [42]^2^
TIPP	25	54.5 ± 13.2	4.93 ± 1.62	2	0	0
LR	25	34 ± 23.5	4.63 ± 2.22	1	0	0
Karatepe *et al.* [43]
TIPP	19	Not investigated	Not investigated	1	0	0
LR	21			0	0	0
Kawji *et al.* [44]^3^
TIPP	21	Not investigated	2.2 ± 1.01	3	0	0
LR	83		2.5 ± 1.9	2	0	0
Koning *et al.* [45]
TIPP	143	34.1 ± 9.9	4.1 ± 1.2	9	5	2
LR	159	39.9 ± 12.0	4.3 ± 1.3	29	20	4
Muldoon *et al.* [46]
TIPP	121	Not investigated	76/79	5/109	10/121	1/121
LR	126		83/86	4/115	9/126	5/126
Nienhuijs *et al.* [47]
TIPP	86	Not investigated	39/78 2.8 ± 2.3	7/86	17/82	2/86
LR	85		50/78 2.8 ± 2.3	12/85	34/84	2/85
Vatansev *et al.* [48]^2^
TIPP	21	51.9 ± 6.5	Not investigated	Not investigated	Not investigated	Not investigated
LR	24	50.7 ± 15.3				

*Standard deviation estimated from the *P*-value.

^1^ Data taken from the published Cochrane review [25]. ^2^ Four arms randomized, controlled trial. Data of TIPP and LR arms was used for combined analysis. ^3^ Five arms randomized, controlled trial. Data of TIPP and LR arms was used for combined analysis.

### Methodological quality of included studies

**Figure 2:**
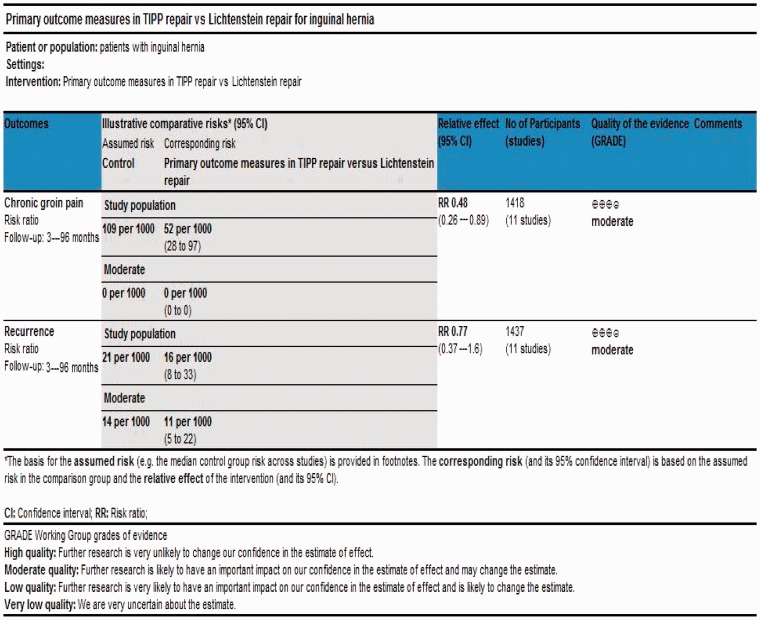
Strength and summary of the evidence analysed on GradePro®.

According to Jadad *et al.* and Chalmers *et al.* [34, 35] the quality of the majority of included trials [37–39, 41–44, 46–48] was low due to the inadequate randomization technique and absence of adequate allocation concealment, power calculations, blinding and intention-to-treat analysis [Table 4]. Based on the quality of included randomized controlled trials, the strength and summary of evidence analysed on GradePro® is given in Figure 2 [36].

**Table 4:** Quality assessment of included trials

Trial	Randomisation technique	Power calculations	Blinding	Intention-to-treat analysis	Allocation Concealment
Berrevoet *et al.* [37]	Computer generated	Not available	No	Yes	Adequate
Dogru *et al.* [38]	Admission order	No	Unclear	No	Adequate
Erhan *et al.* [39]	Admission order	No	No	No	Inadequate
Farooq *et al.* [40]	Computer generated	Yes	Yes	No	Adequate
Gunal *et al.* [41]	Random allocation	No	No	No	Inadequate
Hamza *et al.* [42]	Random number allocation	No	Yes	No	Inadequate
Karatepe *et al.* [43]	Random tables	No	No	No	Adequate
Kawji *et al.* [44]	Unclear	No	No	No	Inadequate
Koning *et al.* [45]	Computer generated	Yes	Yes	Yes	Adequate
Muldoon *et al.* [46]	Computer generated series	No	No	No	Envelope based Adequate
Nienhuijs *et al.* [47]	Computer generated list	No	Yes	No	Adequate
Vatansev *et al.* [48]	Sealed envelops	No	No	No	Adequate

### Primary outcome measures

#### Chronic groin pain

**Figure 3:**
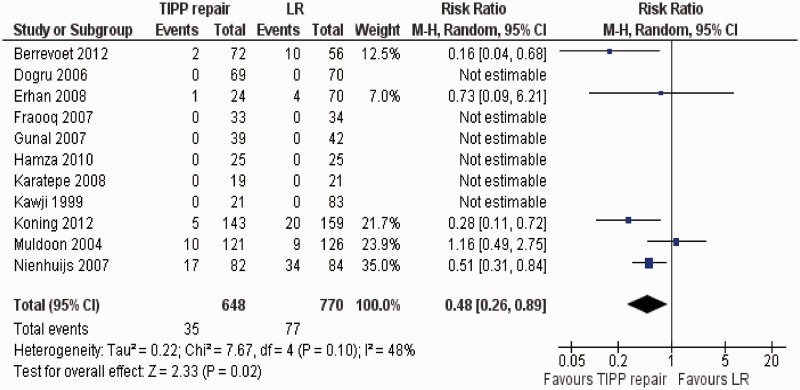
Forest plot for chronic groin pain following TIPP repair vs LR. Risk ratios are shown with 95 per cent confidence intervals. TIPP = transinguinal preperitoneal, LR = Lichtenstein repair.

Eleven randomized, controlled trials [37–47] contributed to the combined calculation of this variable. There was moderate heterogeneity among trials (Tau^2^ = 2.22, chi^2^ = 7.67, df = 4, [*P = *0.10]; I^2^ = 48%). In the random effects model (RR, 0.48; 95% CI, 0.26, 0.89; z = 2.33; *P* < 0.02: Figure 3), the risk of developing chronic groin pain following TIPP repair was lower compared to the use of LR.

#### Recurrence

**Figure 4:**
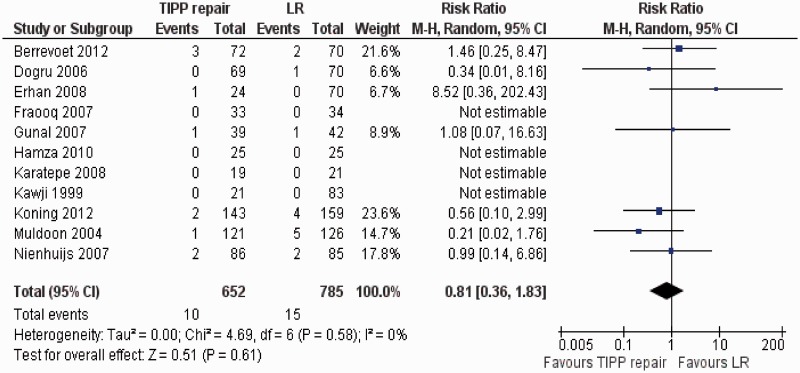
Forest plot for recurrence following TIPP repair vs LR. Risk ratios are shown with 95 per cent confidence intervals. TIPP = transinguinal preperitoneal, LR = Lichtenstein repair.

There was no heterogeneity among trials (Tau^2^ = 0.00, chi^2^ = 4.69, df = 6, [*P* = 0.58]; I^2^ = 0%). In the random effects model (RR, 0.18; 95% CI, 0.36, 1.83; z = 0.51; *P* = 0.61: Figure 4), the risk of developing recurrent inguinal hernia following TIPP repair and LR was statistically similar.

### Secondary outcomes measures

#### Postoperative complications

**Figure 5:**
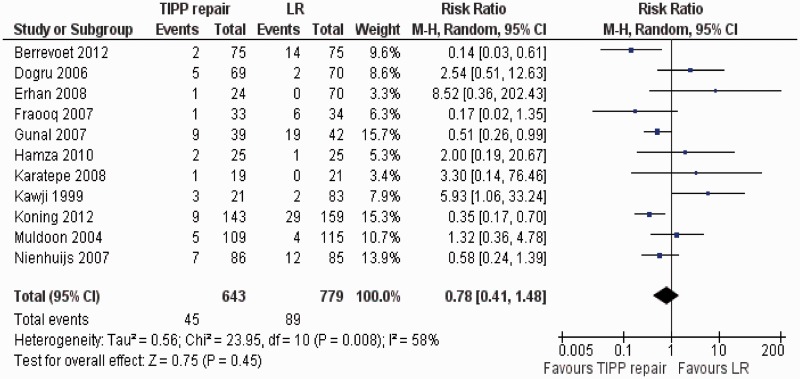
Forest plot for postoperative complications following TIPP repair vs LR. Risk ratios are shown with 95 per cent confidence intervals. TIPP = transinguinal preperitoneal, LR = Lichtenstein repair.

Eleven randomized, controlled trials [37–47] contributed to the combined calculation of this variable. There was moderate heterogeneity (Tau^2^ = 0.56, chi^2^ = 23.95, df = 10, [*P = *0.008]; I^2^ = 58%) among trials. In the random effects model (RR, 0.78; 95% CI, 0.41, 1.48; z = 0.75; *P = *0.45; Figure 5), the risk of developing postoperative complications was statistically similar in both groups.

#### Postoperative incidence of moderate-to-severe pain

**Figure 6:**
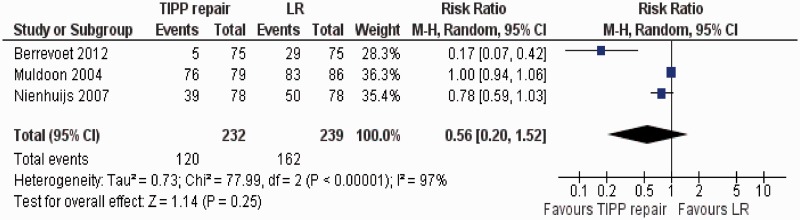
Forest plot for postoperative incidence of moderate to severe pain following TIPP repair vs LR. Risk ratios are shown with 95 per cent confidence intervals. TIPP = transinguinal preperitoneal, LR = Lichtenstein repair.

Three randomized, controlled trials [37, 46, 47] contributed to the combined calculation of this variable. There was significant heterogeneity (Tau^2^ = 0.73; chi^2^ = 77.99, df = 2, [*P* < 0.00001]; I^2^ = 97%) among trials. In the random effects model (RR, 0.56; 95% CI, 0.20, 1.52; z = 1.14; *P = *0.25; Figure 6), the incidence of postoperative moderate-to-severe pain was statistically similar in both groups.

#### Postoperative intensity of pain

**Figure 7:**
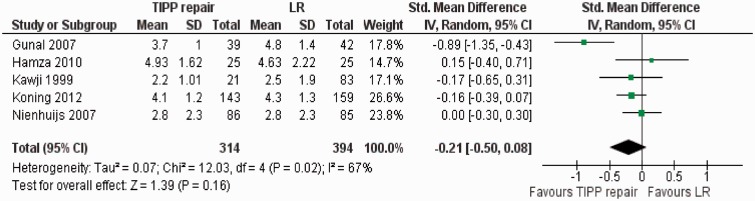
Forest plot for postoperative pain intensity following TIPP repair vs LR. Standardized mean difference (SMD) is shown with 95 per cent confidence intervals. TIPP = transinguinal preperitoneal, LR = Lichtenstein repair.

Five randomized, controlled trials [41, 42, 44, 45, 47] contributed to the combined calculation of this variable. There was significant heterogeneity (Tau^2^ = 0.07; chi^2^ = 12.03, df = 4, [*P* < 0.02]; I^2^ = 67%) among trials. In the random effects model (SMD, −0.21; 95% CI, −0.50, 0.08; z = 1.39; *P = *0.16; Figure 7), the postoperative pain score in both groups was similar.

#### Duration of operation

**Figure 8:**
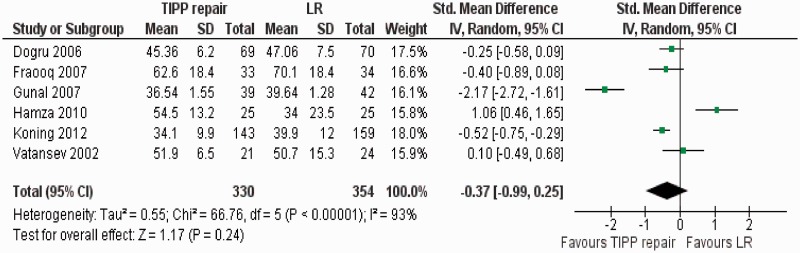
Forest plot for duration of operation following TIPP repair vs LR. Standardized mean difference (SMD) is shown with 95 per cent confidence intervals. TIPP = transinguinal preperitoneal, LR = Lichtenstein repair.

There was significant heterogeneity (Tau^2^ = 0.55; chi^2^ = 66.76, df = 5, [*P* < 0.00001]; I^2^ = 93%) among trials. Therefore, in the random effects model (SMD, −0.37; 95% CI, −0.99, 0.25; z = 1.17; *P = *0.24; Figure 8), the duration of operation for TIPP repair and LR was statistically similar.

## DISCUSSION

This systematic review demonstrates that TIPP repair was associated with a reduced risk of developing chronic groin pain and similar risk of inguinal hernia recurrence, compared to LR. Risk of developing postoperative complications and moderate-to-severe postoperative pain was similar following TIPP repair and LR. In addition, duration of operation was statistically similar in both groups. Findings of this analysis are in concordance with the two previously published systematic reviews [25, 26]. However, these reviews provided limited conclusions, due to methodological flaws and paucity of randomized, controlled trials. Willaert *et al.* [25] reported a meta-analysis of two published and one unpublished, randomized, controlled trials and failed to provide substantial evidence in favour of TIPP repair, due to lack of optimum number of studies and recruited patients [25]. In addition, Li *et al.* [26] reported a systematic review of 12 studies (10 randomized, controlled trials and two comparative studies) which quoted the potential benefits of TIPP in terms of reduced risk of developing chronic groin pain with equivocal postoperative complications and risk of hernia recurrence. But that review also failed to provide a conclusive statement because five included trials were comparing LR against Prolene™ Hernia System leading to potential biases in the inclusion criteria. The present review is reporting the combined conclusion of only those trials which compared the placement of mesh on posterior wall of the inguinal canal against the placement of mesh in the preperitoneal space and, therefore, provides adequate evidence in favour of TIPP repair. TIPP repair may be considered a viable alternative to LR due to its proven advantages in this review.

There are several limitations to the present review. There were significant differences in inclusion and exclusion criteria among the included randomized, controlled trials, such as the recruitment of unilateral inguinal hernia, bilateral inguinal hernia, recurrent inguinal hernia and femoral hernia. Further sub-classification of the inguinal hernia in the form of direct and indirect was also not considered at the time of patient selection. Varying degrees of differences also existed among included randomized, controlled trials regarding the definitions of ‘chronic groin pain’ and ‘measurement scales for postoperative pain’. Randomized, controlled trials [40–43, 48] with fewer patients in this review may not have been sufficient to recognise small differences in outcomes. Included trials with more than two treatment arms [41, 42, 44, 48] may also be considered a biased approach for inclusion. Mesh fixation techniques were a noticeable confounding variable among included trials. Trials in the LR group were not homogenous in terms of mesh fixation technique and, therefore, are potential sources of bias. In addition, in the TIPP group, three studies [39, 40, 46] also reported suture mesh fixation [Table 2] whereas, in the remaining trials in this group, mesh was not fixed. Quality of included trials was poor due to inadequate randomization technique, allocation concealment, power calculations, blinding and intention-to-treat analysis [Table 4]. Variables like foreign body sensation, groin stiffness and decreased groin compliance should have been considered because displaced and rolled-up mesh is likely to cause these symptoms. Our conclusion is based on the summated outcome of 12 randomized, controlled trials but it should be considered with caution because the quality of the majority of included trials was low. There is still a lack of stronger evidence to support the routine use of TIPP repair but it can be considered an alternative and may be applied in selected groups of patients in the beginning. A major, multi-centre, randomized, controlled trial of high quality, according to CONSORT guidelines, is mandatory to validate these findings.

**Conflict of interest:** none declared.
